# Once‐weekly (70 mg/m^2^) vs twice‐weekly (56 mg/m^2^) dosing of carfilzomib in patients with relapsed or refractory multiple myeloma: A post hoc analysis of the ENDEAVOR, A.R.R.O.W., and CHAMPION‐1 trials

**DOI:** 10.1002/cam4.2945

**Published:** 2020-02-28

**Authors:** Philippe Moreau, Keith A. Stewart, Meletios Dimopoulos, David Siegel, Thierry Facon, James Berenson, Noopur Raje, Jesus G. Berdeja, Robert Z. Orlowski, Hui Yang, Haijun Ma, Zandra Klippel, Anita Zahlten‐Kumeli, Khalid Mezzi, Karim Iskander, Maria‐Victoria Mateos

**Affiliations:** ^1^ University Hospital of Nantes Nantes France; ^2^ Mayo Clinic Scottsdale AZ USA; ^3^ School of Medicine National and Kapodistrian University of Athens Athens Greece; ^4^ John Theurer Cancer Center Hackensack University Medical Center Hackensack NJ USA; ^5^ Hôpital Claude Huriez Lille University Hospital Lille France; ^6^ Institute for Myeloma & Bone Cancer Research West Hollywood CA USA; ^7^ Massachusetts General Hospital Cancer Center Boston MA USA; ^8^ Sarah Cannon Research Institute Nashville TN USA; ^9^ The University of Texas MD Anderson Cancer Center Houston TX USA; ^10^ Amgen Inc. Thousand Oaks CA USA; ^11^ University Hospital Salamanca/IBSAL Salamanca Spain

**Keywords:** carfilzomib, dosing schedule, once‐weekly, relapsed and/or refractory multiple myeloma

## Abstract

Combination of carfilzomib with dexamethasone (Kd) is approved for use in relapsed and/or refractory multiple myeloma (RRMM), with carfilzomib administered twice weekly at 56 mg/m^2^ (Kd56 BIW) or once weekly at 70 mg/m^2^ (Kd70 QW). Post hoc cross‐trial comparisons were performed to compare efficacy and safety profiles of Kd70 QW vs Kd56 BIW dosing schedules using data from three trials of patients with RRMM: A.R.R.O.W., CHAMPION‐1, and ENDEAVOR. To select for comparable patient populations, side‐by‐side efficacy and safety comparisons were performed in subgroups of patients with 2‐3 prior lines of therapy who were not refractory to bortezomib. The overall response rate (ORR) was 69.9% (95% confidence interval [CI], 61.7‐77.2) for Kd70 QW and 72.4% (95% CI, 65.9‐78.2) for Kd56 BIW. Median progression‐free survival (PFS) was 12.1 months (95% CI, 8.4‐14.3) for Kd70 QW and 14.5 months (95% CI, 10.2—not evaluable) for Kd56 BIW. Frequency of grade ≥ 3 adverse events (AEs) was 67.6% for Kd70 QW and 85.3% for Kd56 BIW. Regression analyses (adjusting for prognostic factors) of all patients in the trials who received Kd70 QW vs Kd56 BIW estimated a PFS hazard ratio of 0.91 (95% CI, 0.69‐1.19; *P* = .47) and an ORR odds ratio of 1.12 (95% CI, 0.74‐1.69; *P* = .61). These results suggest that Kd70 QW has a comparable efficacy profile compared with Kd56 BIW and represents a convenient and well‐tolerated treatment for patients with RRMM.

## Graphical abstract

In this study, we performed post hoc cross‐trial comparisons to compare efficacy and safety profiles of carfilzomib with dexamethasone, administered once weekly at 70 mg/m^2^ (Kd70 QW) vs Kd 56 mg/m^2^, administered twice weekly (Kd56 BIW) using pooled data from three trials of patients with relapsed and/or refractory multiple myeloma (RRMM). Our results suggest that Kd70 QW has a comparable efficacy profile compared with Kd56 BIW and represents a convenient and well‐tolerated treatment for patients with RRMM.

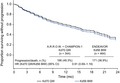

## 1 INTRODUCTION

Carfilzomib is a selective second‐generation proteasome inhibitor that is approved for the treatment of patients with relapsed and/or refractory multiple myeloma (RRMM).^1^, ^2^ In the United States, carfilzomib was initially approved as a single agent to treat patients with advanced multiple myeloma. As the benefit‐risk profile of carfilzomib became better understood, different doses, dosing schedules, and combination regimens were explored. Carfilzomib was subsequently approved for use in RRMM in combination with dexamethasone (Kd) with once‐ and twice‐weekly dosing options.^3^, ^4^

The twice‐weekly Kd regimen was approved in 2016 with carfilzomib dosed at 56 mg/m^2^ in combination with dexamethasone at 20 mg per dose (Kd56 BIW). This approval was based on the ENDEAVOR trial, a randomized phase 3 trial of patients with RRMM who had 1‐3 prior lines of therapy. ENDEAVOR demonstrated superior progression‐free survival (PFS) and overall survival with Kd56 BIW compared with bortezomib in combination with dexamethasone.^5^, ^6^

The more convenient once‐weekly Kd dosing schedule was first explored in CHAMPION‐1, a phase 1/2 dose‐finding study of Kd in patients with RRMM. The maximum tolerated dose (MTD) of once‐weekly carfilzomib was 70 mg/m^2^ in combination with dexamethasone at 40 mg weekly (Kd70 QW). The overall response rate (ORR) and median PFS at the MTD were comparable with previous studies of twice‐weekly dosing.^7^, ^8^, ^9^

Subsequently, Kd70 QW was formally assessed in the phase 3 A.R.R.O.W. trial of patients with RRMM, which compared Kd70 QW vs twice‐weekly Kd with 27 mg/m^2^ carfilzomib (Kd27 BIW). Kd70 QW significantly prolonged PFS and ORR vs Kd27 BIW, with a similar safety profile.^10^ Based on the outcomes from A.R.R.O.W., Kd70 QW was approved in 2018 in the United States.

To date, Kd70 QW and Kd56 BIW have not been directly compared in a randomized, head‐to‐head trial. We performed a post hoc analysis of data from the ENDEAVOR, CHAMPION‐1, and A.R.R.O.W. trials for a side‐by‐side comparison of efficacy and safety profiles of Kd70 QW with Kd56 BIW.

## 2 METHODS

### 2.1 Patients and study design

Data from three previously described trials of carfilzomib in RRMM were analyzed (A.R.R.O.W. [ClinicalTrials.gov identifier: NCT02412878], CHAMPION‐1 [NCT01677858], and ENDEAVOR [NCT01568866]).^5^, ^9^, ^10^ The Kd70 QW data used in this analysis were obtained from the A.R.R.O.W. and CHAMPION‐1 studies, and the Kd56 BIW data were obtained from the ENDEAVOR study. The study design and eligibility criteria of each study have been previously reported in detail.^5^, ^9^, ^10^ Briefly, the phase 3 ENDEAVOR study was a head‐to‐head comparison of carfilzomib and bortezomib, both combined with dexamethasone at 20 mg per dose, for patients with RRMM with 1‐3 prior lines of therapy. In this study, carfilzomib was administered twice weekly at 56 mg/m^2^. Patients with prior bortezomib or carfilzomib treatment were eligible provided they achieved at least a partial response to the treatment, were not discontinued due to tolerability issues, and had a ≥6‐month interval free of proteasome inhibitor treatment before enrollment.^5^ The primary endpoint was PFS and secondary endpoints included ORR, safety, and overall survival. CHAMPION‐1 was a phase 1/2 dose‐finding study of once‐weekly administration of carfilzomib in patients with RRMM with 1‐3 prior lines of therapy (patients with prior carfilzomib therapy were excluded).^9^ The MTD of once‐weekly carfilzomib was 70 mg/m^2^ in combination with dexamethasone at 40 mg also once weekly (Kd70 QW). Efficacy endpoints were ORR and PFS, which were determined for all patients treated at the MTD from phases 1 and 2 of the study. Lastly, A.R.R.O.W. was a phase 3 open‐label, randomized trial that compared carfilzomib administered once weekly at 70 mg/m^2^ vs twice weekly at 27 mg/m^2^ (the only approved carfilzomib dose at the time of the study design and most of the enrollment period) with dexamethasone at 40 mg once weekly. Patients included in A.R.R.O.W. had RRMM with 2‐3 previous therapies and were refractory to the most recent therapy.^10^ The primary endpoint was PFS and secondary endpoints included ORR, safety, and overall survival.

In A.R.R.O.W., 240 patients were randomized to receive Kd70 QW; in CHAMPION‐1, 104 patients received Kd70 QW; and in ENDEAVOR, 464 patients were randomized to receive Kd56 BIW. All patients provided written informed consent and all study protocols were approved by the institutional review boards or ethics committees of participating institutions.

### 2.2 Outcomes

Efficacy endpoints of this post hoc analysis included ORR and PFS with the Kd70 QW and Kd56 BIW treatment modalities. Overall survival was not assessed in this post hoc analysis due to lack of complete collection of survival data in the CHAMPION‐1 study. For the safety analysis, the rate of treatment‐emergent adverse events (AEs), including the rate of serious AEs and AEs of interest, was assessed for patients receiving Kd70 QW and Kd56 BIW. To control for variances in eligibility criteria and reported baseline clinical characteristics across the three studies, side‐by‐side efficacy and safety comparisons between the Kd70 QW and Kd56 BIW cohorts were performed with subgroups of patients who received 2‐3 prior lines of therapy and were not refractory to bortezomib. The Kd70 QW subgroups from A.R.R.O.W. and CHAMPION‐1 were pooled together and compared with the Kd56 BIW subgroup from ENDEAVOR.

### 2.3 Statistical analysis

Data cutoff dates were as follows: A.R.R.O.W. (20 July 2017), CHAMPION‐1 (30 August 2016), and ENDEAVOR (31 January 2015 for PFS and ORR; 8 February 2017 for safety). Exact 95% confidence intervals (CIs) for ORR were determined based on binomial distribution. Medians for PFS were estimated using the Kaplan‐Meier method. The 95% CIs for PFS medians were estimated using the log‐log transformation method by Klein and Moeschberger.^11^ Median follow‐up time was estimated using the reverse Kaplan‐Meier method and 95% CIs were estimated using the log‐log transformation method by Klein and Moeschberger.^11^, ^12^

Regression analyses were performed to further assess the relationship between the Kd schedules and efficacy. The regression models included data from all 808 patients who received treatment with Kd70 QW or Kd56 BIW from the three trials (this analysis was performed independently of the subgroup analyses described above). Cox and logistic regression models were used to estimate PFS hazard ratios and ORR odds ratios, respectively, with 95% CIs. Prognostic covariates entered into the models included age (<65, 65 to <75, and ≥75 years), International Staging System (ISS) stage (1 vs 2 and 3), bortezomib‐refractory status (yes vs no), lenalidomide‐refractory status (yes vs no), and number of prior lines of therapy (1‐2 vs 3). Due to the high proportion of patients with unknown cytogenetic risk (in particular, in the A.R.R.O.W. study), cytogenetic risk was not included as a covariate. The models quantified the relationship between Kd70 QW vs Kd56 BIW treatment for PFS and ORR after adjusting for these prognostic factors. For all analyses, descriptive *P*‐values are presented as appropriate without adjustment for multiplicity.

## 3 RESULTS

Due in part to variances in eligibility criteria among the CHAMPION‐1, A.R.R.O.W., and ENDEAVOR trials, there were differences across the patient populations enrolled in these studies. Therefore, to study safety and efficacy of Kd56 BIW vs Kd70 QW in a more comparable patient population, we performed analyses in subgroups of patients who had received 2‐3 prior lines of therapy and were not refractory to bortezomib. Side‐by‐side analyses of efficacy and safety were performed on pooled data from 146 patients who received Kd70 QW in A.R.R.O.W. and CHAMPION‐1 and compared with the 217 patients who received Kd56 BIW in ENDEAVOR. Baseline patient characteristics of Kd70 QW vs Kd56 BIW subgroups were generally balanced; however, differences in proportions of patients who received prior treatment with bortezomib or lenalidomide were observed (Table 1).

**Table 1:** Baseline patient characteristics of Kd70 QW and Kd56 BIW subgroups (patients with 2‐3 prior lines of therapy and not refractory to prior bortezomib)

Patient characteristics	Kd70 QW	Kd56 BIW
	A.R.R.O.W. + CHAMPION‐1 (n = 146)	ENDEAVOR (n = 217)
Sex		
Male	79 (54.1)	112 (51.6)
Female	67 (45.9)	105 (48.4)
Age group, y		
Mean (SD)	64.9 (9.7)	63.9 (9.7)
<65	68 (46.6)	112 (51.6)
65 to <75	53 (36.3)	74 (34.1)
≥75	25 (17.1)	31 (14.3)
ISS stage		
1	65 (44.5)	97 (44.7)
2 and 3	80 (54.8)	120 (55.3)
Missing	1 (0.7)	0
Total number of prior therapies		
2	84 (57.5)	148 (68.2)
3	62 (42.5)	69 (31.8)
Prior treatment with bortezomib	139 (95.2)	139 (64.1)
Prior treatment with lenalidomide	128 (87.7)	117 (53.9)
Refractory to lenalidomide	116 (79.5)	83 (38.2)
ECOG performance status		
0	73 (50.0)	104 (47.9)
1‐2	73 (50.0)	113 (52.1)
Creatinine clearance,^a^ mL/min		
Mean (SD)	83.1 (37.9)	79.1 (33.9)
Median (range)	76.7 (27.6‐257.7)	75.0 (14.0‐182.0)

Note

Data are n (%) unless specified otherwise.

Abbreviations: ECOG, Eastern Cooperative Oncology Group; ISS, International Staging System; Kd56 BIW, twice‐weekly carfilzomib dosed at 56 mg/m^2^ in combination with a standard dexamethasone dose; Kd70 QW, once‐weekly carfilzomib dosed at 70 mg/m^2^ in combination with a standard dexamethasone dose; SD, standard deviation.

^a^ Creatinine clearance was calculated using the Cockcroft‐Gault formula.

**FIGURE 1:**
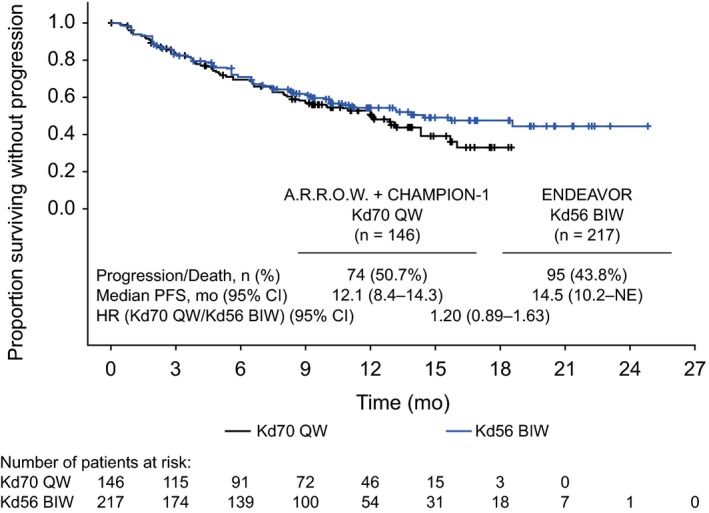
PFS of Kd70 QW and Kd56 BIW subgroups (patients with 2‐3 prior lines of therapy and not refractory to prior bortezomib). CI, confidence interval; HR, hazard ratio; Kd56 BIW, twice‐weekly carfilzomib dosed at 56 mg/m^2^ in combination with a standard dexamethasone dose; Kd70 QW, once‐weekly carfilzomib dosed at 70 mg/m^2^ in combination with a standard dexamethasone dose; NE, not evaluable; PFS, progression‐free survival

In the side‐by‐side subgroups comparison, ORR and PFS were similar between Kd70 QW and Kd56 BIW. The ORR was 69.9% (95% CI, 61.7‐77.2) for Kd70 QW (n = 146) and 72.4% (95% CI, 65.9‐78.2) for Kd56 BIW (n = 217; Table S1). Complete response or better was achieved in 8.2% of patients receiving Kd70 QW and 13.3% of patients receiving Kd56 BIW. Median PFS was 12.1 months (95% CI, 8.4‐14.3) for Kd70 QW and 14.5 months (95% CI, 10.2—not evaluable) for Kd56 BIW (Figure 1). Median follow‐up time for PFS was 12.9 months (95% CI, 11.4‐13.8) for Kd70 QW and 11.2 months (95% CI, 10.2‐13.0) for Kd56 BIW. The mean (standard deviation) relative dose intensity was 92.7% (10.9) for Kd70 QW compared with 87.0% (14.5) for Kd56 BIW.

In subgroups of patients who had received 2‐3 prior lines of therapy and were not refractory to bortezomib, the overall frequency of grade ≥ 3 treatment‐emergent AEs was 67.6% with Kd70 QW (n = 145) and 85.3% with Kd56 BIW (n = 217; Table 2). For AEs of interest, the rate of grade ≥ 3 AEs in Kd70 QW and Kd56 BIW, respectively, was 1.4% and 5.1% for cardiac failure, 3.4% and 6.0% for acute renal failure, 2.1% and 2.3% for embolic and thrombotic events, and 5.5% and 15.7% for hypertension. Median time of treatment exposure was similar for the Kd70 QW (38.1 weeks; range, 0.1‐82.4) and Kd56 BIW (40.3 weeks; range, 0.3‐210.0) subgroups. The proportion of patients with AEs leading to treatment discontinuation was also similar between Kd70 QW (10.3%) and Kd56 BIW (13.8%). At the end of the first 6 months, the rate of grade ≥ 3 AEs was 56.6% for Kd70 QW and 68.7% for Kd56 BIW (Table S2).

**Table 2:** AEs in Kd70 QW and Kd56 BIW subgroups (patients with 2‐3 prior lines of therapy and not refractory to prior bortezomib)

	Kd70 QW	Kd56 BIW
	A.R.R.O.W. + CHAMPION‐1 (n = 145)	ENDEAVOR (n = 217)
All treatment‐emergent adverse events, n (%)	140 (96.6)	217 (100.0)
Grade ≥ 3	98 (67.6)	185 (85.3)
Serious adverse events	57 (39.3)	141 (65.0)
Cardiac failure^a^	2 (1.4)	19 (8.8)
Grade ≥ 3	2 (1.4)	11 (5.1)
Acute renal failure^a^	9 (6.2)	22 (10.1)
Grade ≥ 3	5 (3.4)	13 (6.0)
Embolic and thrombotic events, venous^a^	5 (3.4)	23 (10.6)
Grade ≥ 3	3 (2.1)	5 (2.3)
Hypertension^a^	27 (18.6)	69 (31.8)
Grade ≥ 3	8 (5.5)	34 (15.7)
Adverse events leading to discontinued treatment	15 (10.3)^b^	30 (13.8)

Abbreviations: AE, adverse event; Kd56 BIW, twice‐weekly carfilzomib dosed at 56 mg/m^2^ in combination with a standard dexamethasone dose; Kd70 QW, once‐weekly carfilzomib dosed at 70 mg/m^2^ in combination with a standard dexamethasone dose; MedDRA, Medical Dictionary for Regulatory Activities.

^a^ Standardized MedDRA Queries Narrow terms.

^b^ Analysis set of n = 146 was used to determine AEs leading to discontinued treatment in the Kd70 QW subgroup.

**FIGURE 2:**
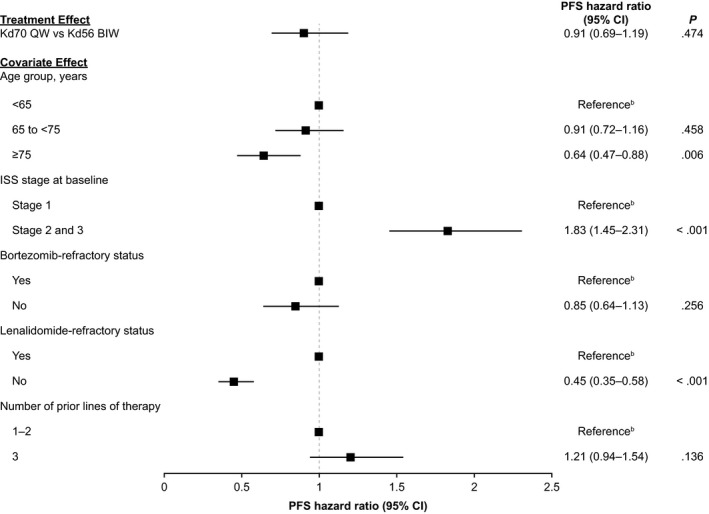
Regression analysis results of PFS of Kd70 QW vs Kd56 BIW^a^. ^a^All 808 patients with observed data receiving Kd70 QW in the A.R.R.O.W. (n = 240) and CHAMPION‐1 (n = 104) trials and Kd56 BIW in the ENDEAVOR (n = 464) trial. Includes treatment group, age, ISS stage, bortezomib‐refractory status, lenalidomide‐refractory status, and number of prior lines of therapy as covariates. ^b^Reference group (hazard ratio = 1.0). Covariate effect refers to the effect of the corresponding category vs reference category of the variable. CI, confidence interval; ISS, International Staging System; Kd56 BIW, twice‐weekly carfilzomib dosed at 56 mg/m^2^ in combination with a standard dexamethasone dose; Kd70 QW, once‐weekly carfilzomib dosed at 70 mg/m^2^ in combination with a standard dexamethasone dose; PFS, progression‐free survival

**FIGURE 3:**
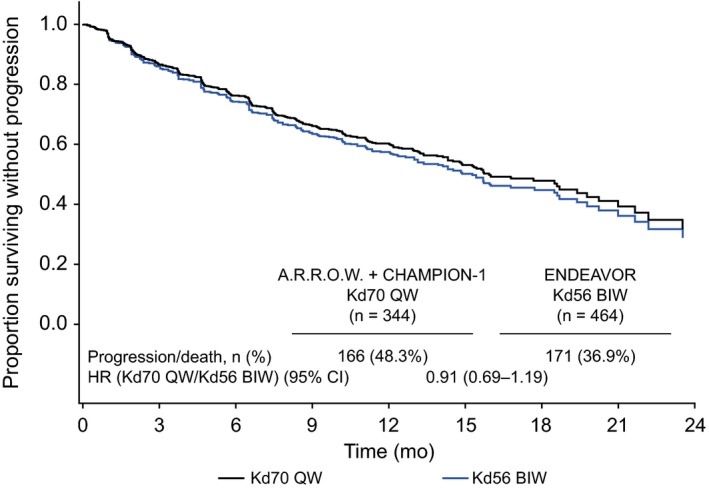
Multiple Cox regression analysis results of PFS of Kd70 QW and Kd56 BIW^a^. ^a^All 808 patients with observed data receiving Kd70 QW in the A.R.R.O.W. (n = 240) and CHAMPION‐1 (n = 104) trials and Kd56 BIW in the ENDEAVOR (n = 464) trial. Direct‐adjusted survival curves derived from the Cox regression model with treatment group, age, ISS stage, bortezomib‐refractory status, lenalidomide‐refractory status, and number of prior lines of therapy as covariates. CI, confidence interval; HR, hazard ratio; Kd56 BIW, twice‐weekly carfilzomib dosed at 56 mg/m^2^ in combination with a standard dexamethasone dose; Kd70 QW, once‐weekly carfilzomib dosed at 70 mg/m^2^ in combination with a standard dexamethasone dose; PFS, progression‐free survival

**FIGURE 4:**
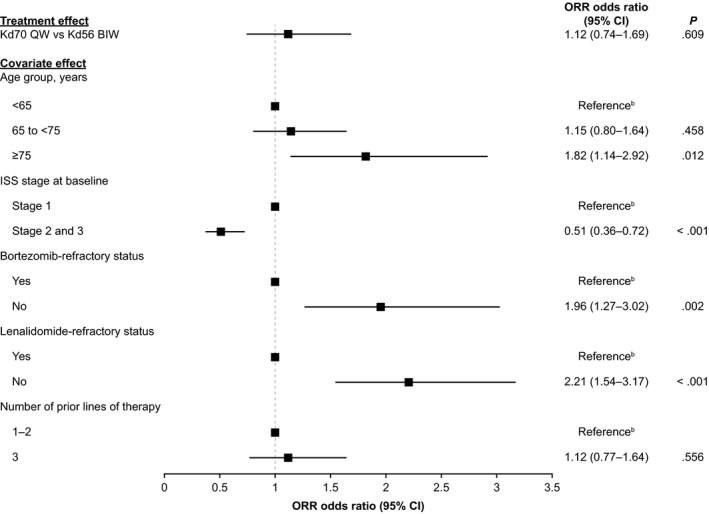
Regression analysis results of ORR of Kd70 QW vs Kd56 BIW^a^. ^a^All 808 patients with observed data receiving Kd70 QW in the A.R.R.O.W. (n = 240) and CHAMPION‐1 (n = 104) trials and Kd56 BIW in the ENDEAVOR (n = 464) trial. Includes treatment group, age, ISS stage, bortezomib‐refractory status, lenalidomide‐refractory status, and number of prior lines of therapy as covariates. ^b^Reference group (odds ratio = 1.0). Covariate effect refers to the effect of the corresponding category vs reference category of the variable. CI, confidence interval; ISS, International Staging System; Kd56 BIW, twice‐weekly carfilzomib dosed at 56 mg/m^2^ in combination with a standard dexamethasone dose; Kd70 QW, once‐weekly carfilzomib dosed at 70 mg/m^2^ in combination with a standard dexamethasone dose; ORR, overall response rate

Multiple Cox proportional hazards regression model analyses were performed on all 808 patients who received Kd70 QW (n = 344) or Kd56 BIW (n = 464) in the three trials. There was no statistically significant difference between Kd70 QW vs Kd56 BIW for PFS after adjusting for prognostic covariates (hazard ratio, 0.91; 95% CI, 0.69‐1.19; *P* = .47; Figures 2 and 3), and results remained the same with a random study effect term added to the model (hazard ratio, 0.91; 95% CI, 0.69‐1.19; *P* = .47). Age group, ISS stage, and lenalidomide‐refractory status were associated with PFS with *P* < .05 in the model. Similarly, there was no significant difference in ORR for Kd70 QW vs Kd56 BIW after adjusting for prognostic covariates in the multivariate logistic regression model, with an odds ratio of 1.12 (95% CI, 0.74‐1.69; *P* = .61; Figure 4). Adding a random study effect term to the model resulted in a similar odds ratio of 1.12 (95% CI, 0.71‐1.77; *P* = .62). Age, ISS stage, bortezomib‐refractory status, and lenalidomide‐refractory status were associated with ORR with *P* < .05.

## 4 DISCUSSION

Kd70 QW and Kd56 BIW dosing schedules are both approved for treatment of RRMM, but they have not been directly compared in a randomized trial. Here, we performed a post hoc analysis of pooled data from the A.R.R.O.W., CHAMPION‐1, and ENDEAVOR trials for an adjusted side‐by‐side comparison of efficacy and safety outcomes for Kd70 QW and Kd56 BIW in patients with RRMM. We conducted our analyses in a subset of patients from the three trials to control for potential confounding factors in eligibility criteria and baseline patient characteristics across the trials. Considerably fewer bortezomib‐refractory patients were enrolled in the Kd56 BIW ENDEAVOR arm (n = 15; 3%) compared with the Kd70 QW arms of A.R.R.O.W. (n = 111; 46%) and CHAMPION‐1 (n = 54; 52%). Additionally, patients in ENDEAVOR and CHAMPION‐1 had relapsed or refractory myeloma and received 1‐3 prior lines of therapy, but enrollment in A.R.R.O.W. was limited to patients with refractory myeloma and 2‐3 prior lines of therapy. Therefore, in this post hoc analysis, we selected subgroups of patients from each trial who were non‐refractory to prior bortezomib treatment and had received 2‐3 lines of prior therapy for side‐by‐side comparison.

Side‐by‐side comparisons of the subgroups revealed that the ORR and median PFS were similar for Kd70 QW vs Kd56 BIW dosing. To further address the potential of confounding results due to differences in patient populations, the impact of known prognostic covariates was assessed in multivariate analyses. Prognostic factors included age, ISS stage, number of prior lines of therapy, and bortezomib‐ or lenalidomide‐refractory status. After adjusting for these prognostic covariates, the results of regression modeling further supported the side‐by‐side findings; there was no significant difference in efficacy outcomes between the Kd70 QW and Kd56 BIW dosing schedules.

In the side‐by‐side analysis of the subgroups, fewer grade ≥ 3 AEs were observed with Kd70 QW (67.6%) compared with Kd56 BIW (85.3%), despite similar median treatment exposure times (38.1 vs 40.3 weeks). The frequency of grade ≥ 3 AEs of interest also differed between Kd70 QW and Kd56 BIW, with lower rates of cardiac failure (1.4% vs 5.1%), acute renal failure (3.4% vs 6.0%), and hypertension (5.5% vs 15.7%) observed with Kd70 QW vs Kd56 BIW. In a safety analysis limited to the first 6 months from receiving treatment, a lower frequency of grade ≥ 3 AEs with Kd70 QW vs Kd56 BIW was also observed. These results support that Kd70 QW represents a convenient and well‐tolerated dosing option for patients with RRMM.

Cross‐trial comparisons are inherently difficult to make because a number of factors (eg, study design, disease and patient heterogeneity, and disease‐ and treatment‐related factors) have the potential to confound direct comparisons.^13^, ^14^ Eligibility criteria across multiple myeloma trials often differ and can result in an imbalance between trial populations.^13^, ^14^ Treatment outcomes can also be affected by various disease and patient characteristics, such as patient age, ISS stage, and prior exposure to therapies, which can further confound cross‐trial comparisons.^14^, ^15^, ^16^, ^17^, ^18^, ^19^ For patients with RRMM who have been exposed to multiple therapies, the number of prior therapies and refractory status to prior therapies can considerably influence outcomes to additional treatments.^20^, ^21^, ^22^ With the increased use of lenalidomide and proteasome inhibitors in the upfront setting and lenalidomide as maintenance therapy, the incidence of patients who become refractory to immunomodulatory drugs and proteasome inhibitors is increasing, which can also significantly affect study outcomes.^22^, ^23^ These factors were carefully considered in the design of the post hoc side‐by‐side cross‐trial analyses and adjusted for in regression models. However, cytogenetic risk was not included in our analyses due to the high proportion of patients with unknown cytogenetic risk in the parent studies.

In this post hoc cross‐trial comparison, we utilized independent methods to compare the efficacy and safety of Kd70 QW and Kd56 BIW dosing regimens in patients with RRMM. The efficacy with Kd70 QW and Kd56 BIW dosing was comparable and their safety profiles were similar, with slightly lower frequencies of grade ≥ 3 AEs observed with Kd70 QW. Altogether, our findings suggest that Kd70 QW represents a convenient dosing schedule with a favorable benefit‐risk profile for the treatment of patients with RRMM.

## AUTHOR CONTRIBUTIONS

All authors played a role in the interpretation and analysis of data, in drafting the manuscript, and in the decision to submit the manuscript for publication.

## Data Availability

Qualified researchers may request data from Amgen clinical studies. Complete details are available at the following: http://www.amgen.com/datasharing
