# Supplementary material for: Once‐weekly (70 mg/m2) vs twice‐weekly (56 mg/m2) dosing of carfilzomib in patients with relapsed or refractory multiple myeloma: A post hoc analysis of the ENDEAVOR, A.R.R.O.W., and CHAMPION‐1 trials
Source: Cancer Med. 2020 Feb 28;9(9):2989–96. doi: 10.1002/cam4.2945 (PMC7196059; doi:10.1002/cam4.2945)
Supplement: Supplementary file 1 [file CAM4-9-2989-s001.docx]

**Table S1. ORR of Kd70 QW and Kd56 BIW subgroups (patients with 2–3 prior lines of therapy and not refractory to prior bortezomib)**

|  | **Kd70 QW** | **Kd56 BIW** |
| --- | --- | --- |
|  | **A.R.R.O.W. + CHAMPION-1 (n = 146)** | **ENDEAVOR (n = 217)** |
| Best overall response, n (%) |  |  |
| Stringent complete response | 4 (2.7) | 2 (0.9) |
| Complete response | 8 (5.5) | 27 (12.4) |
| Very good partial response | 50 (34.2) | 72 (33.2) |
| Partial response | 40 (27.4) | 55 (25.3) |
| Overall response rate (95% CI) | 69.9  (61.7–77.2) | 72.4  (65.9–78.2) |

CI, confidence interval; Kd56 BIW, twice-weekly carfilzomib dosed at 56 mg/m^2^ in combination with a standard dexamethasone dose; Kd70 QW, once-weekly carfilzomib dosed at 70 mg/m^2^ in combination with a standard dexamethasone dose; ORR, overall response rate.
