# Supplementary material for: Once‐weekly (70 mg/m2) vs twice‐weekly (56 mg/m2) dosing of carfilzomib in patients with relapsed or refractory multiple myeloma: A post hoc analysis of the ENDEAVOR, A.R.R.O.W., and CHAMPION‐1 trials
Source: Cancer Med. 2020 Feb 28;9(9):2989–96. doi: 10.1002/cam4.2945 (PMC7196059; doi:10.1002/cam4.2945)
Supplement: Supplementary file 2 [file CAM4-9-2989-s002.docx]

**Table S2. Six-month AEs of Kd70 QW and Kd56 BIW subgroups (patients with 2–3 prior lines of therapy and not refractory to prior bortezomib)**

|  | **Kd70 QW** | **Kd56 BIW** |
| --- | --- | --- |
|  | **A.R.R.O.W. + CHAMPION-1 (n = 145)** | **ENDEAVOR (n = 217)** |
| Grade ≥ 3 | 82 (56.6) | 149 (68.7) |
| Serious adverse events | 48 (33.1) | 80 (36.9) |
| Cardiac failure^a^ | 2 (1.4) | 8 (3.7) |
| Grade ≥ 3 | 2 (1.4) | 5 (2.3) |
| Acute renal failure^a^ | 8 (5.5) | 11 (5.1) |
| Grade ≥ 3 | 4 (2.8) | 6 (2.8) |
| Embolic and thrombotic events, venous^a^ | 4 (2.8) | 14 (6.5) |
| Grade ≥ 3 | 3 (2.1) | 4 (1.8) |
| Hypertension^a^ | 16 (11.0) | 41 (18.9) |
| Grade ≥ 3 | 4 (2.8) | 14 (6.5) |

^a^Standardized MedDRA Queries Narrow terms.

AE, adverse event; Kd56 BIW, twice-weekly carfilzomib dosed at 56 mg/m^2^ in combination with a standard dexamethasone dose; Kd70 QW, once-weekly carfilzomib dosed at 70 mg/m^2^ in combination with a standard dexamethasone dose; MedDRA, Medical Dictionary for Regulatory Activities.
